# Supplementary material for: Investigation of the anti-tumor mechanism of tirabrutinib, a highly selective Bruton’s tyrosine kinase inhibitor, by phosphoproteomics and transcriptomics
Source: PLoS One. 2023 Mar 10;18(3):e0282166. doi: 10.1371/journal.pone.0282166 (PMC10004634; doi:10.1371/journal.pone.0282166)
Supplement: S1 Fig — (A) TMD8 and (B) U-2932 cells were treated with vehicle or different concentrations of tirabrutinib and incubated for 4 h at 37°C (5% CO2/95% air). Autophosphorylated BTK (p-BTK) (upper) and total BTK (BTK) (lower) proteins were detected by western blot analysis. TMD8 cells were unstimulated (‒) or stimulated using H2O2 and used as a marker for the detection of p-BTK and BTK. (PDF) [file pone.0282166.s001.pdf]

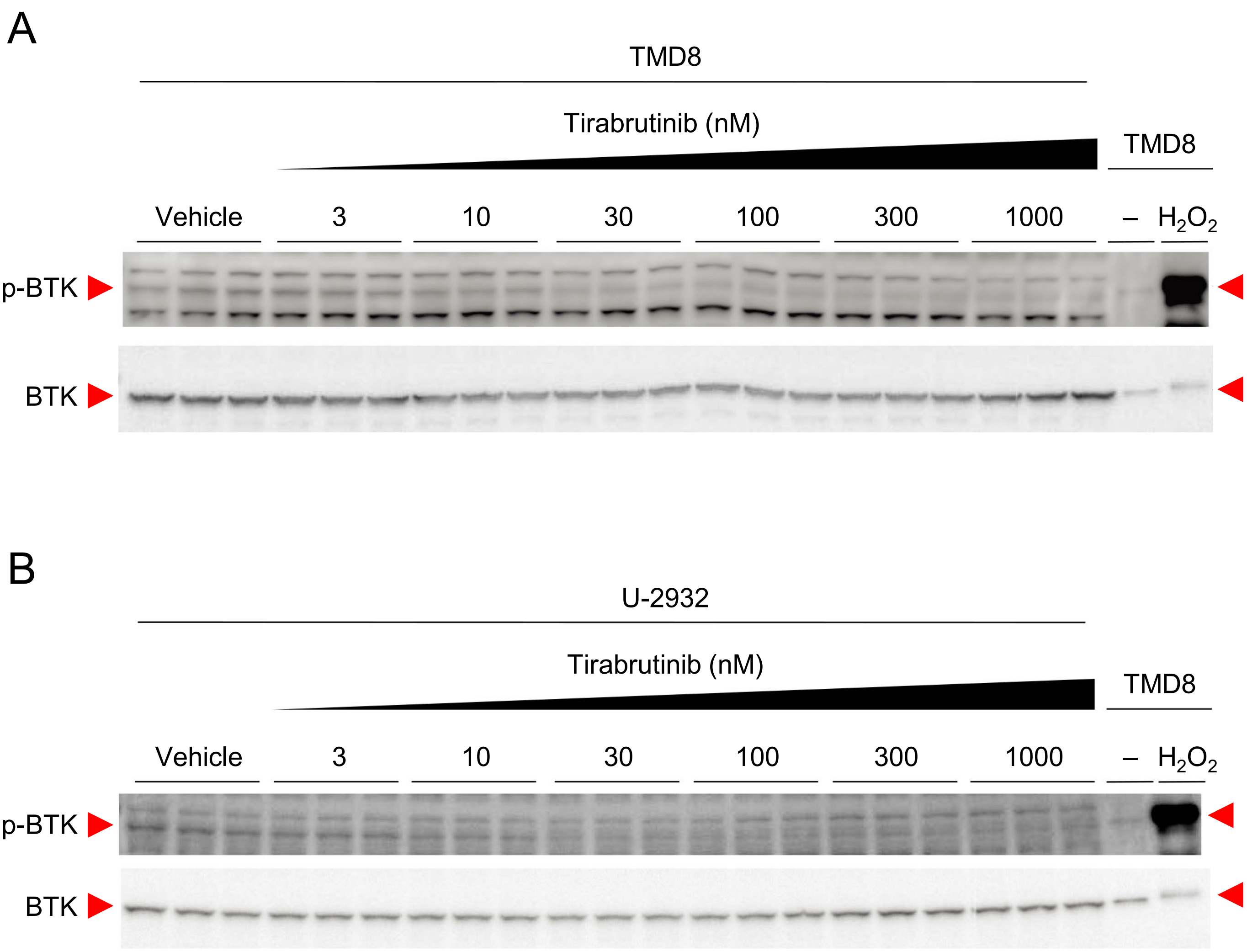


**S1 Figure. Immunoblots of lysates to evaluate BTK autophosphorylation inhibitory effects of tirabrutinib in the TMD8 and U-2932 cell lines.**

(**A**) TMD8 and (**B**) U-2932 cells were treated with vehicle or different concentrations of tirabrutinib and incubated for 4 h at 37°C (5% $CO_2$/95% air). Autophosphorylated BTK (p-BTK) (upper) and total BTK (BTK) (lower) proteins were detected by western blot analysis. TMD8 cells were unstimulated (–) or stimulated using $H_2O_2$ and used as a marker for the detection of p-BTK and BTK.
