# Supplementary material for: Investigation of the anti-tumor mechanism of tirabrutinib, a highly selective Bruton’s tyrosine kinase inhibitor, by phosphoproteomics and transcriptomics
Source: PLoS One. 2023 Mar 10;18(3):e0282166. doi: 10.1371/journal.pone.0282166 (PMC10004634; doi:10.1371/journal.pone.0282166)
Supplement: S2 Fig — (A) TMD8 cells or (B) U-2932 cells were treated with tirabrutinib (10, 30, 100, or 300 nM) or DMSO and incubated for 1 and/or 4 h at 37°C in 5% CO2/95% air. Autophosphorylated BTK (p-BTK, Tyr-223), total BTK (BTK), phosphorylated PLCγ2 (p-PLCγ2, Tyr-759), total PLCγ2 (PLCγ2), phosphorylated ERK1/2 (p-ERK, Thr-202/204), total ERK1/2, and GAPDH proteins were detected by western blot analysis. TMD8 cells were stimulated using H2O2 and used as a marker for the detection of p-BTK, p-PLCγ2, and p-ERK. (PDF) [file pone.0282166.s002.pdf]

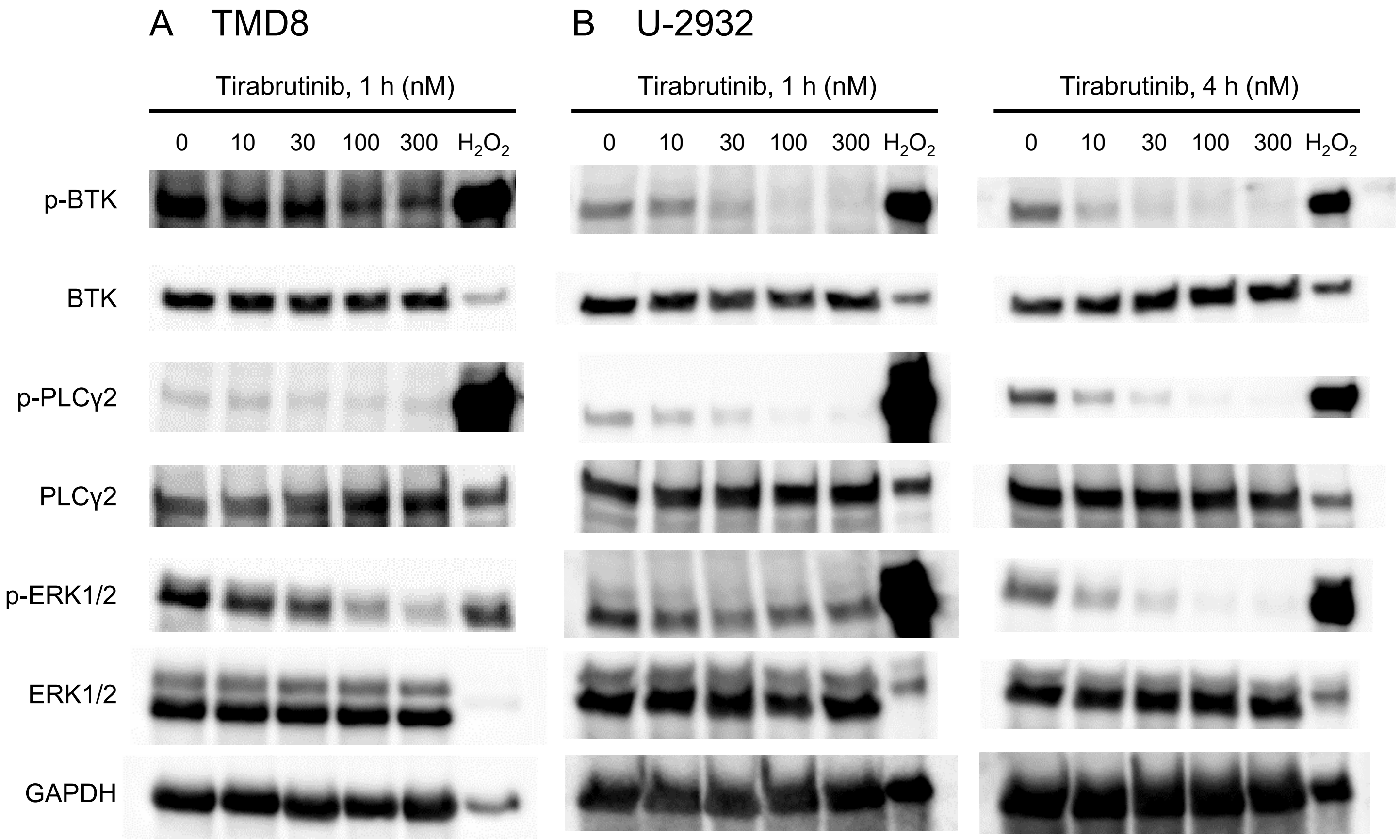

S2 Figure. Immunoblots for p-BTK, BTK, p-PLCγ2, PLCγ2, p-ERK1/2, ERK1/2, and GAPDH in lysates of TMD8 and U-2932 cells treated with or without tirabrutinib (0–300 nM).

(**A**) TMD8 cells or (**B**) U-2932 cells were treated with tirabrutinib (10, 30, 100, or 300 nM) or DMSO and incubated for 1 and/or 4 h at 37°C in 5% CO_2/95% air. Autophosphorylated BTK (p-BTK, Tyr-223), total BTK (BTK), phosphorylated PLCγ2 (p-PLCγ2, Tyr-759), total PLCγ2 (PLCγ2), phosphorylated ERK1/2 (p-ERK, Thr-202/204), total ERK1/2, and GAPDH proteins were detected by western blot analysis. TMD8 cells were stimulated using H_2O_2 and used as a marker for the detection of p-BTK, p-PLCγ2, and p-ERK.
