# Supplementary material for: Investigation of the anti-tumor mechanism of tirabrutinib, a highly selective Bruton’s tyrosine kinase inhibitor, by phosphoproteomics and transcriptomics
Source: PLoS One. 2023 Mar 10;18(3):e0282166. doi: 10.1371/journal.pone.0282166 (PMC10004634; doi:10.1371/journal.pone.0282166)
Supplement: S3 Fig — U-2932 cells and TMD8 were treated with tirabrutinib (1 μM) or DMSO and incubated for 1 or 4 h at 37°C in 5% CO2/95% air. (A) Phosphorylated AKT (p-AKT, Ser-473) and total AKT (AKT) proteins were detected by western blot analysis. (B) Phosphorylated IκBα (p-IκBα, Ser-32/36), total IκBα (IκBα), and GAPDH proteins were detected by western blot analysis. (PDF) [file pone.0282166.s003.pdf]

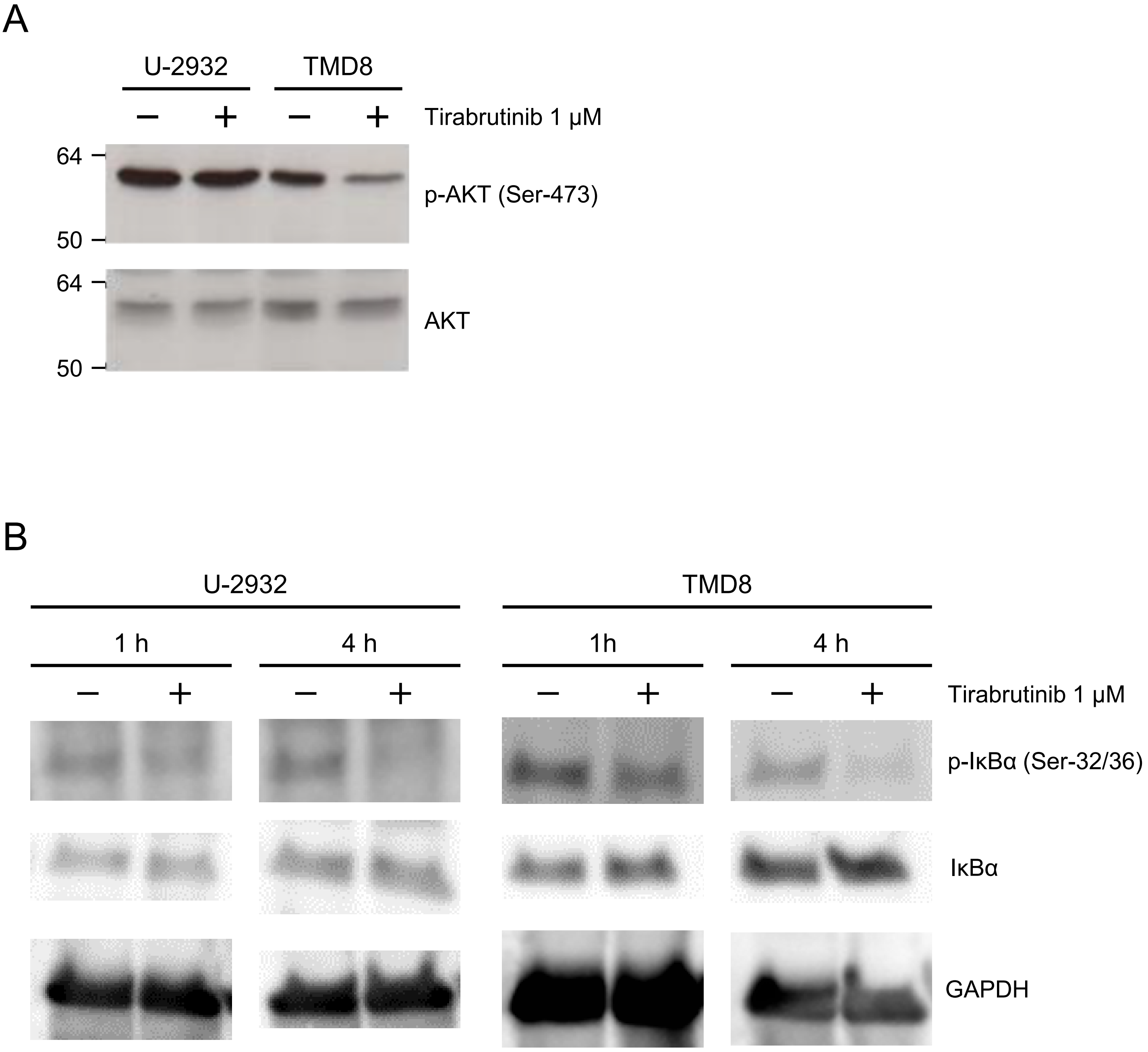

S3 Figure. Immunoblots of p-AKT, AKT, p-IκBα, IκBα, and GAPDH in lysates of TMD8 and U-2932 cells treated with or without tirabrutinib (1 μM).

U-2932 cells and TMD8 were treated with tirabrutinib (1 μM) or DMSO and incubated for 1 or 4 h at 37°C in 5% CO_2/95% air. (**A**) Phosphorylated AKT (p-AKT, Ser-473) and total AKT (AKT) proteins were detected by western blot analysis. (**B**) Phosphorylated IκBα (p-IκBα, Ser-32/36), total IκBα (IκBα), and GAPDH proteins were detected by western blot analysis.
