# Supplementary figures and images for: Investigation of the anti-tumor mechanism of tirabrutinib, a highly selective Bruton’s tyrosine kinase inhibitor, by phosphoproteomics and transcriptomics
Source: PLoS One. 2023 Mar 10;18(3):e0282166. doi: 10.1371/journal.pone.0282166 (PMC10004634; doi:10.1371/journal.pone.0282166)

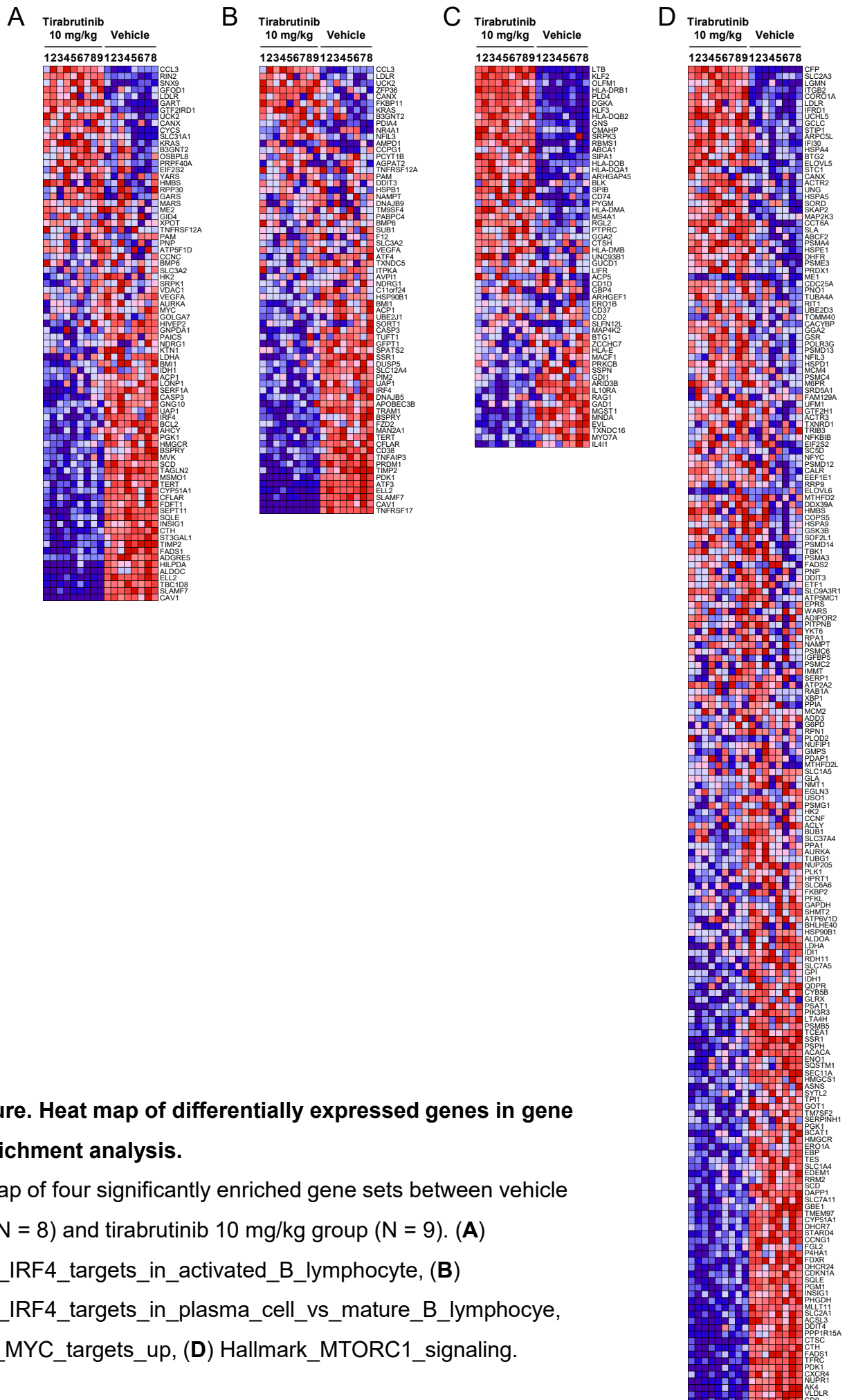

Supplement: S4 Fig — Heat map of four significantly enriched gene sets between vehicle group (N = 8) and tirabrutinib 10 mg/kg group (N = 9). (A) Shaffer_IRF4_targets_in_activated_B_lymphocyte, (B) Shaffer_IRF4_targets_in_plasma_cell_vs_mature_B_lymphocye, (C) YU_MYC_targets_up, (D) Hallmark_MTORC1_signaling. (PDF) [file pone.0282166.s004.pdf]

S3 Figure A\_Original Image\_Marker

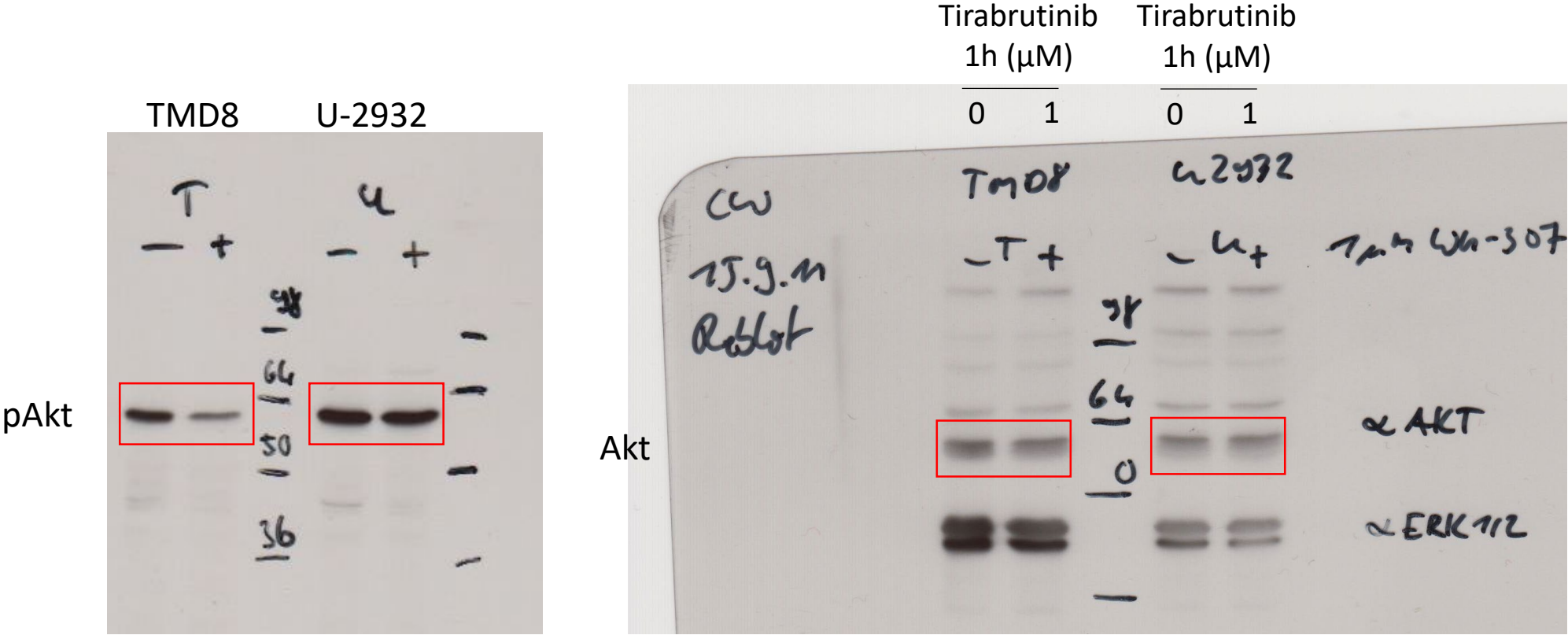

S3 Figure B\_Original Image\_Marker

U2932

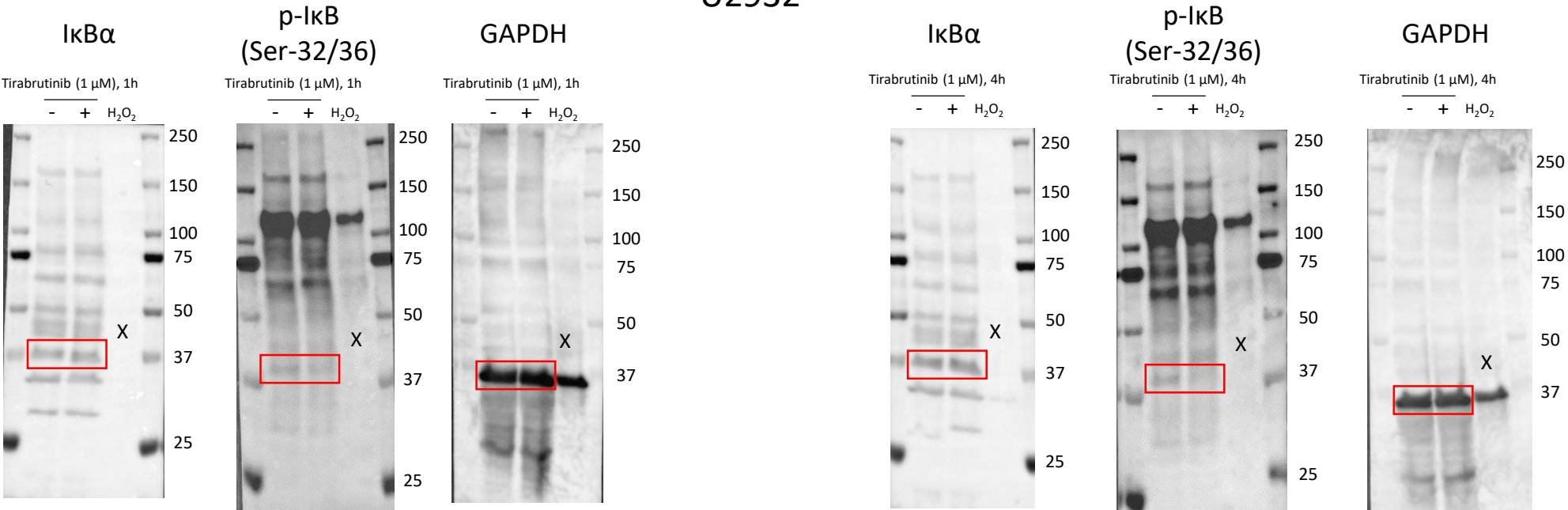

TMD8

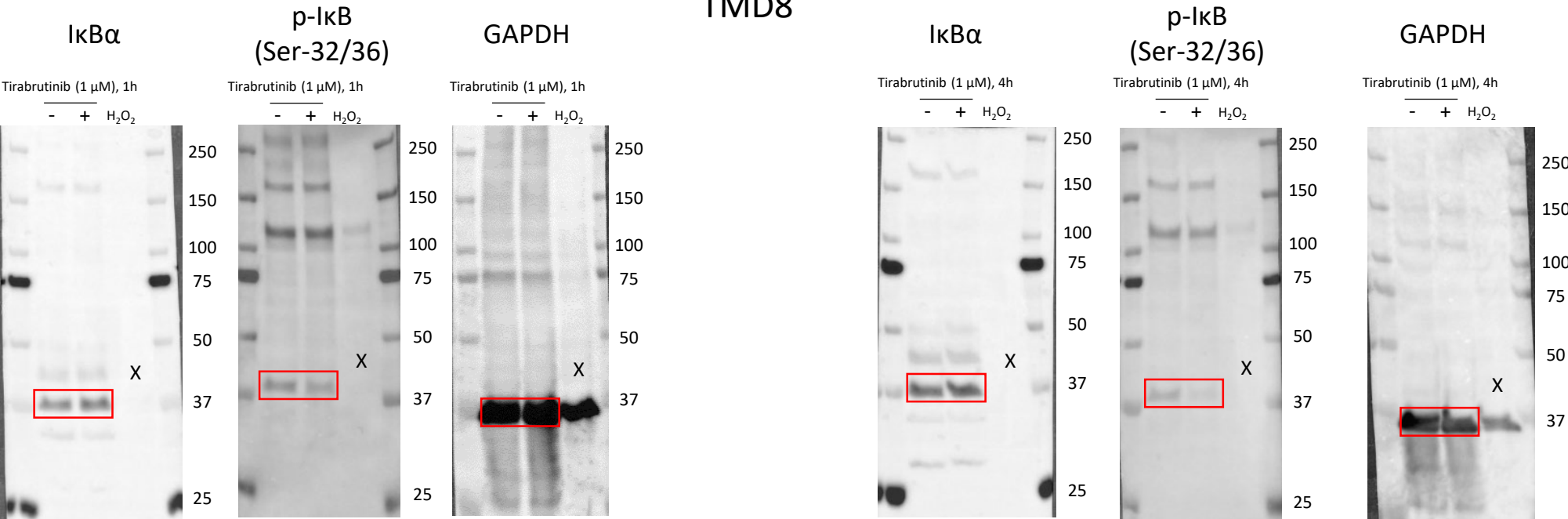

Supplement: S3 Raw images — (PDF) [file pone.0282166.s017.pdf]

S6 Figure B\_Original Image\_Marker

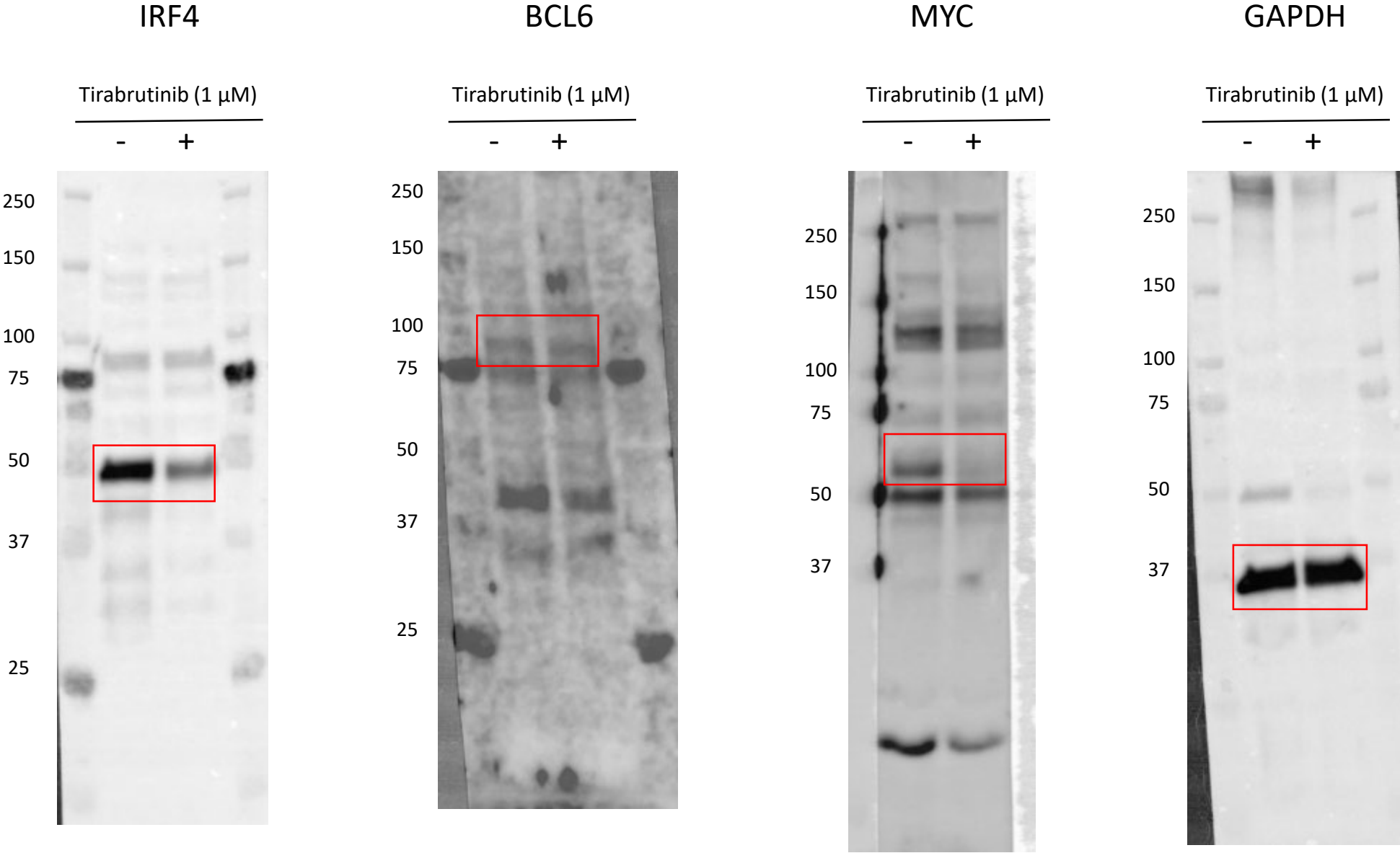

Supplement: S4 Raw images — (PDF) [file pone.0282166.s018.pdf]
