# Supplementary material for: Investigation of the anti-tumor mechanism of tirabrutinib, a highly selective Bruton’s tyrosine kinase inhibitor, by phosphoproteomics and transcriptomics
Source: PLoS One. 2023 Mar 10;18(3):e0282166. doi: 10.1371/journal.pone.0282166 (PMC10004634; doi:10.1371/journal.pone.0282166)
Supplement: S5 Fig — (PDF) [file pone.0282166.s005.pdf]

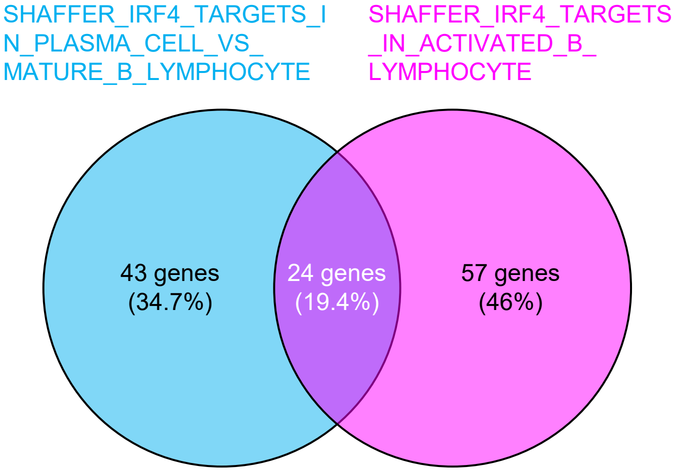

20 genes

AGPAT2	NFIL3
AMPD1	NR4A1
APOBEC3B	PABPC4
ATF3	PCYT1B
ATF4	PDIA4
AVPI1	PDK1
C11orf24	PIM2
CCPG1	PRDM1
CD38	SLC12A4
DDIT3	SORT1
DNAJB5	SPATS2
DNAJB9	SSR1
DUSP5	SUB1
F12	TM9SF4
FKBP11	TNFAIP3
FZD2	TNFRSF17
GFPT1	TRAM1
HSP90B1	TUFT1
HSPB1	TXNDC5
ITPKA	UBE2J1
MAN2A1	ZFP36
NAMPT	

Overlapped 10 genes

ACP1	KRAS
B3GNT2	LDLR
BMI1	NDRG1
BMP6	PAM
BSPRY	SLAMF7
CANX	SLC3A2
CASP3	TERT
CAV1	TIMP2
CCL3	TNFRSF12A
CFLAR	UAP1
ELL2	UCK2
IRF4	VEGFA

19 genes

ADGRE5	KTN1
AHCY	LDHA
ALDOC	LONP1
ATP5F1D	MARS1
AURKA	ME2
BCL2	MSMO1
CCNC	MVK
CTH	MYC
CYCS	OSBPL8
CYP51A1	PAICS
EIF2S2	PGK1
FADS1	PNP
FDFT1	PRPF40A
GARS1	RIN2
GART	RPP30
GFOD1	SCD
GID4	SEPTIN11
GNG10	SERF1A
GNPDA1	SLC31A1
GOLGA7	SNX9
GRSF1	SQLE
GTF2IRD1	SRPK1
HILPDA	ST3GAL1
HIVEP2	TAGLN2
HK2	TBC1D8
HMBS	VDAC1
HMGCR	XPOT
IDH1	YARS1
INSIG1	

Red: Core enrichment genes

S5 Figure. Core enrichment genes of gene set enrichment analysis for interferon regulatory factor target genes in the two gene sets.
