# Supplementary material for: Investigation of the anti-tumor mechanism of tirabrutinib, a highly selective Bruton’s tyrosine kinase inhibitor, by phosphoproteomics and transcriptomics
Source: PLoS One. 2023 Mar 10;18(3):e0282166. doi: 10.1371/journal.pone.0282166 (PMC10004634; doi:10.1371/journal.pone.0282166)
Supplement: S6 Fig — TMD8 were treated with tirabrutinib (1 μM) or DMSO and incubated for 24 h at 37°C in 5% CO2/95% air. (A) RNA was isolated and polymerase chain reaction (PCR) was performed. All PCRs were performed in triplicate, and the mRNA expression of GAPDH was used as an internal control. The t test was used to compare mRNA expression in the DMSO- and tirabrutinib-treated groups. A P-value of less than 5% was considered statistically significant. **: P < 0.01, ***: P < 0.001. (B) IRF4, BCL6, MYC, and GAPDH proteins were detected by western blot analysis. (PDF) [file pone.0282166.s006.pdf]

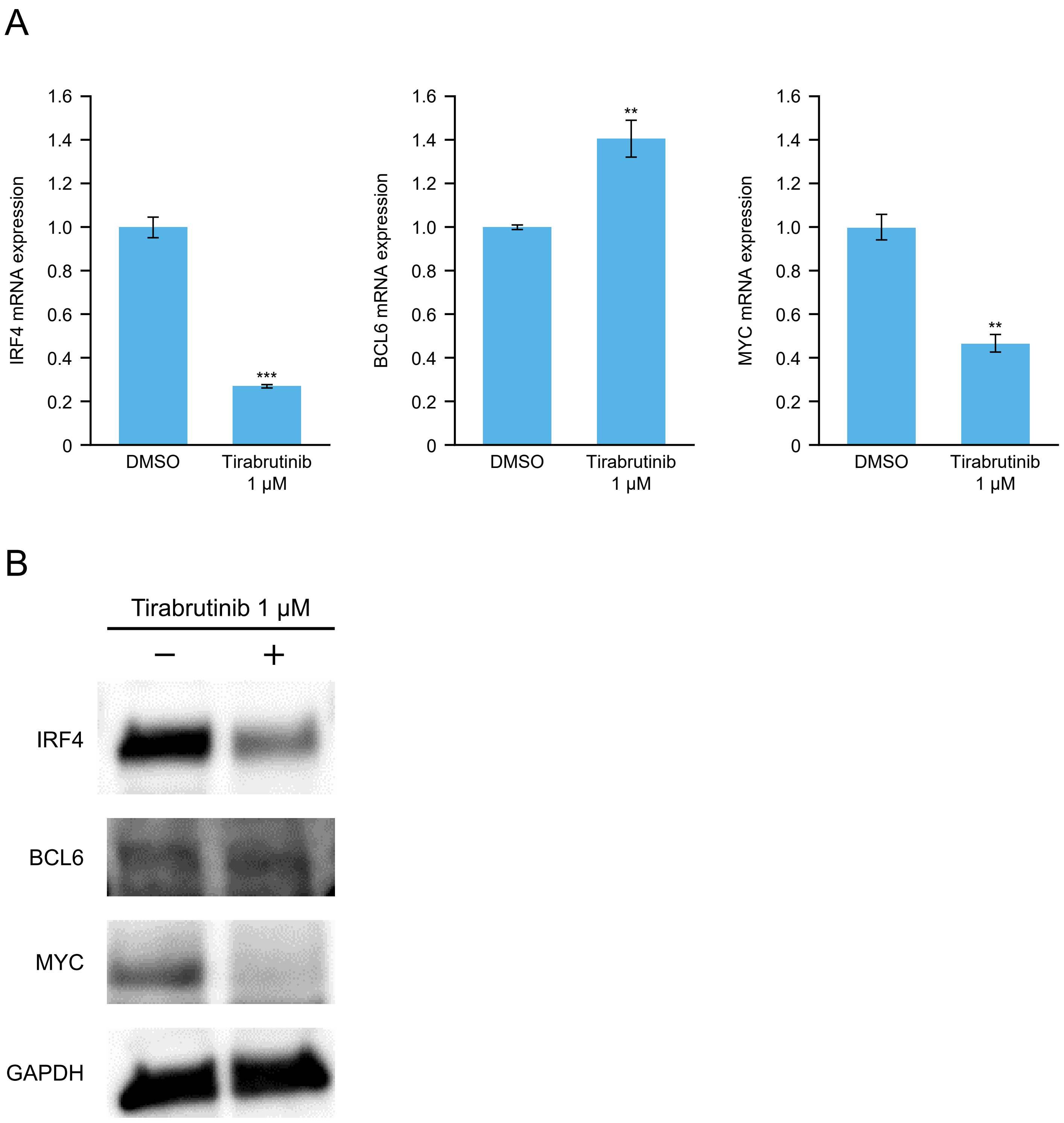

S6 Figure. Gene and protein expression in TMD8 cells treated with or without tirabrutinib. TMD8 were treated with tirabrutinib (1 μM) or DMSO and incubated for 24 h at 37°C in 5% CO_2/95% air. (**A**) RNA was isolated and polymerase chain reaction (PCR) was performed. All PCRs were performed in triplicate, and the mRNA expression of GAPDH was used as an internal control. The *t* test was used to compare mRNA expression in the DMSO- and tirabrutinib-treated groups. A *P*-value of less than 5% was considered statistically significant. **: $P < 0.01$, ***: $P < 0.001$. (**B**) IRF4, BCL6, MYC, and GAPDH proteins were detected by western blot analysis.
