# Supplementary material for: Investigation of the anti-tumor mechanism of tirabrutinib, a highly selective Bruton’s tyrosine kinase inhibitor, by phosphoproteomics and transcriptomics
Source: PLoS One. 2023 Mar 10;18(3):e0282166. doi: 10.1371/journal.pone.0282166 (PMC10004634; doi:10.1371/journal.pone.0282166)
Supplement: S7 Fig — TMD8 were treated with tirabrutinib (1 μM), BMS-345541 (10 μM), MK-2206 (1 μM), refametinib (0.1 μM) or DMSO and incubated for 24 h at 37°C in 5% CO2/95% air. RNA was isolated and polymerase chain reaction (PCR) was performed. All PCRs were performed in triplicate, and the mRNA expression of GAPDH was used as an internal control. The Dunnett test was used to compare mRNA expression in the DMSO- and compound-treated groups. A P-value of less than 5% was considered statistically significant. *: P < 0.05, ***: P < 0.001. n.s.: Not significant. (PDF) [file pone.0282166.s007.pdf]

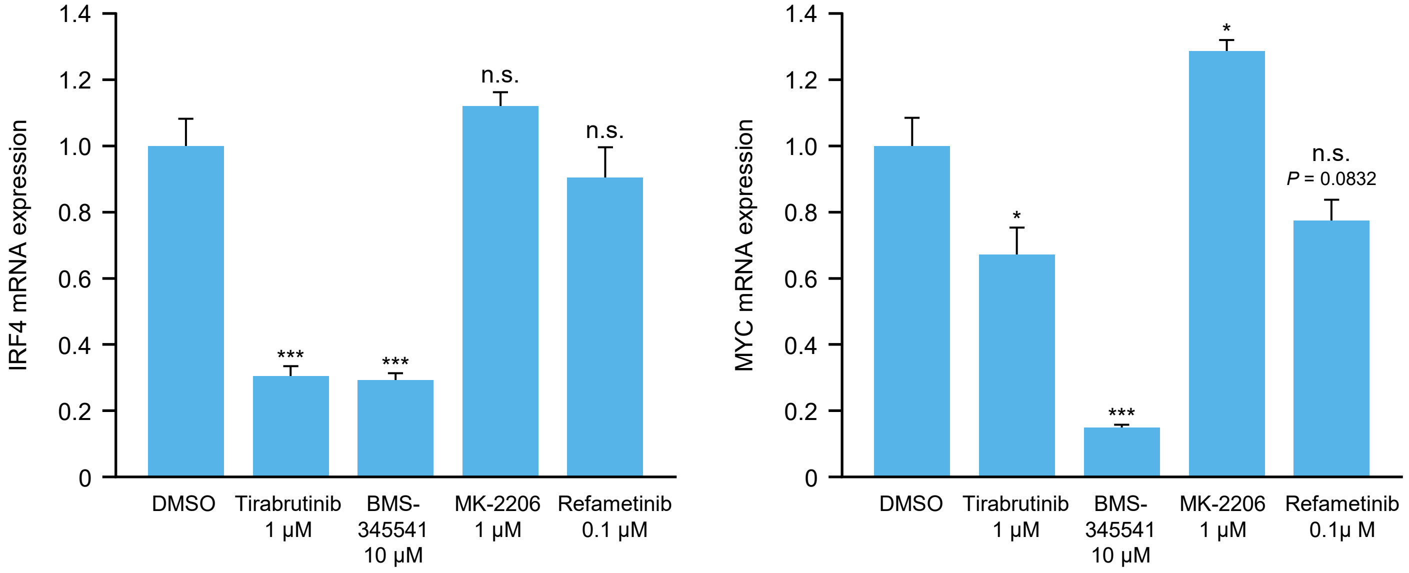

S7 Figure. Gene expression detected by RT-PCR in TMD8 cells treated with or without various selective inhibitors.

TMD8 were treated with tirabrutinib (1 μM), BMS-345541 (10 μM), MK-2206 (1 μM), refametinib (0.1 μM), or DMSO and incubated for 24 h at 37°C in 5% CO_2/95% air. RNA was isolated and polymerase chain reaction (PCR) was performed. All PCRs were performed in triplicate, and the mRNA expression of GAPDH was used as an internal control. The Dunnett test was used to compare mRNA expression in the DMSO- and compound-treated groups. A *P*-value of less than 5% was considered statistically significant. *: $P < 0.05$, ***: $P < 0.001$. n.s.: not significant.
