# Supplementary material for: Investigation of the anti-tumor mechanism of tirabrutinib, a highly selective Bruton’s tyrosine kinase inhibitor, by phosphoproteomics and transcriptomics
Source: PLoS One. 2023 Mar 10;18(3):e0282166. doi: 10.1371/journal.pone.0282166 (PMC10004634; doi:10.1371/journal.pone.0282166)
Supplement: S1 Table — (DOCX) [file pone.0282166.s008.docx]

**S1 Table. The 12 *in vitro* systems of the BioMAP Diversity PLUS panel (Eurofins DiscoverX Products, LLC).**

| **System** | | **Cell type** | **Disease** | **Readouts** |
| --- | --- | --- | --- | --- |
| 3C | 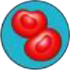 | Venular endothelial cells | Cardiovascular disease, chronic inflammation | CCL2/MCP-1, CD106/VCAM-1, CD141/thrombomodulin, CD142/tissue factor, CD54/ICAM-1, CD62E/E-selectin, CD87/uPAR, CXCL8/lL-8, CXCL9/MIG, HLA-DR, proliferation, SRB |
| 4H | 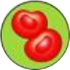 | Venular endothelial cells | Allergy, asthma, autoimmunity | CCL2/MCP-1, CCL26/eotaxin-3, CD106/VCAM-1, CD62P/P-selectin, CD87/uPAR, SRB, VEGFR2 |
| LPS | 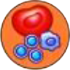 | Peripheral blood　mononuclear cells, venular endothelial cells | Cardiovascular disease, chronic inflammation | CCL2/MCP-1, CD106/VCAM-1, CD141/thrombomodulin, CD142/tissue factor, CD40, CD62E/E-selectin, CD69, CXCL8/lL-8, IL-1 alpha, M-CSF, sPGE2, SRB, sTNF-alpha |
| SAg | 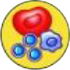 | Peripheral blood　mononuclear cells, venular endothelial cells | Autoimmune disease, chronic inflammation | CCL2/MCP-1, CD38, CD40, CD62E/E-selectin, CD69, CXCL8/1L-8, CXCL9/MIG, PBMC cytotoxicity, proliferation, SRB |
| BT | 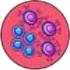 | B cells, peripheral blood mononuclear cells | Allergy, asthma, autoimmunity, oncology | B cell proliferation, PBMC cytotoxicity, secreted IgG, sIL-17A, sIL-17F, sIL-2, sIL-6, sTNF-alpha |
| BF4T | 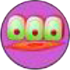 | Bronchial epithelial cells, dermal fibroblasts | Allergy, asthma, fibrosis, lung inflammation | CCL2/MCP-1, CCL26/eotaxin-3, CD106/VCAM-1, CD54/ICAM-1, CD90, CXCL8/IL-8, IL-1 alpha, keratin 8/18, MMP-1, MMP-3, MMP-9, PAI-I, SRB, tPA, uPA |
| BE3C | 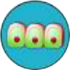 | Bronchial epithelial cells | COPD, lung inflammation | CD54/ICAM-1, CD87/uPAR, CXCL 10/IP-10, CXCL 11/I-TAC, CXCL8/IL-8, CXCL9/MIG, EGFR, HLA-DR, IL-1 alpha, keratin 8/18, MMP-1, MMP-9, PAI-I, SRB, tPA, uPA |
| CASM3C | 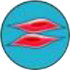 | Coronary artery smooth muscle cells | Cardiovascular inflammation, restenosis | CCL2/MCP-1, CD106/VCAM-1, CD141/thrombomodulin, CD142/tissue factor, CD87/uPAR, CXCL8/1L-8, CXCL9/MIG, HLA-DR, IL-6, LDLR, M-CSF, PAI-I, proliferation, serum amyloid A, SRB |
| HDF3CGF | 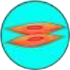 | Dermal fibroblasts | Chronic inflammation, fibrosis | CCL2/MCP-1, CD106/VCAM-1, CD54/ICAM-1, collagen I, collagen Ill, CXCL10/IP-10, CXCL11/I-TAC, CXCL8/IL-8, CXCL9/MIG, EGFR, M-CSF, MMP-1, PAI-I, proliferation_72hr, SRB, TIMP-1, TIMP-2 |
| KF3CT | 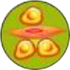 | Dermal fibroblasts, keratinocytes | Dermatitis, psoriasis | CCL2/MCP-1, CD54/ICAM-1, CXCLl0/IP-10, CXCL8/IL-8, CXCL9/MIG, IL-1 alpha, MMP-9, PAI-I, SRB, TIMP-2, uPA |
| MyoF | 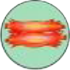 | Lung fibroblasts | Chronic inflammation, fibrosis, matrix remodeling, wound healing | alpha-SM actin, bFGF, CDl06/VCAM-1, collagen I, collagen III, collagen IV, CXCL8/IL-8, decorin, MMP-1, PAI-I, SRB, TIMP-1 |
| *I*Mphg | 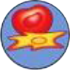 | Macrophages, venular endothelial cells | Cardiovascular disease, chronic inflammation, restenosis | CCL2/MCP-1, CCL3/MIP-1 alpha, CD106/VCAM-1, CD40, CD62E/E-selectin, CD69, CXCL8/IL-8, IL-1 alpha, M-CSF, sIL-10, SRB, SRB-Mphg |

Table of the 12 systems in the BioMAP Diversity PLUS Panel, including a list of the cell types, disease context and list of biomarker readouts optimized for each system. Biomarker endpoint measurements are cell-surface levels unless indicated by the prefix “s”, which denotes soluble levels of biomarker endpoints measured in the system supernatant.

COPD, chronic obstructive pulmonary disease

Reprinted with modification from Singer JW *et al.* PLoS One. 2019:14(9):e0222944 under a CC BY license.
