# Supplementary material for: Investigation of the anti-tumor mechanism of tirabrutinib, a highly selective Bruton’s tyrosine kinase inhibitor, by phosphoproteomics and transcriptomics
Source: PLoS One. 2023 Mar 10;18(3):e0282166. doi: 10.1371/journal.pone.0282166 (PMC10004634; doi:10.1371/journal.pone.0282166)

S2 Figure A_Original Image_Marker (TMD8_Tirabrutinib_1 h)

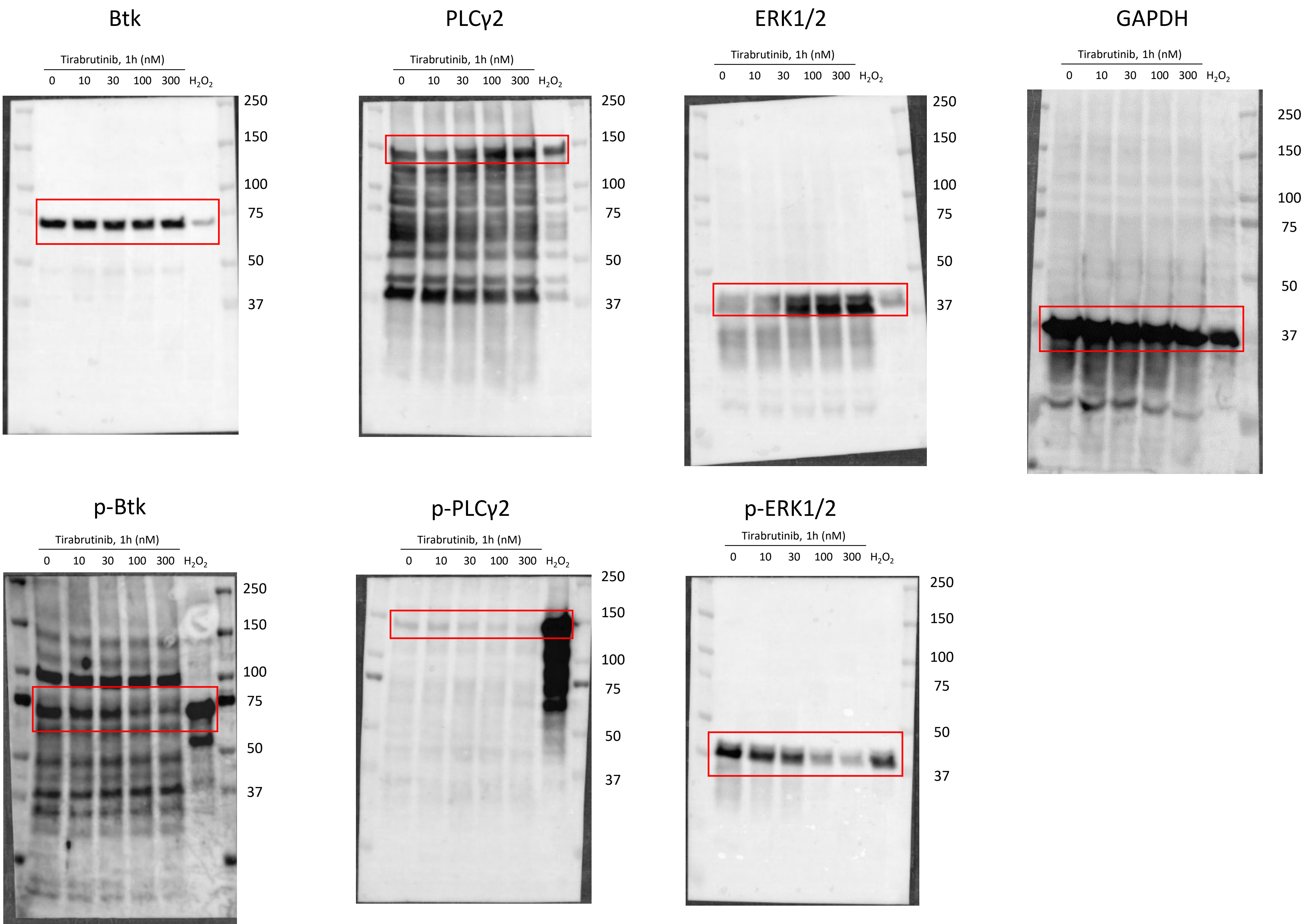

S2 Figure B_Original Image_Marker (U2932_Tirabrutinib_1 h)

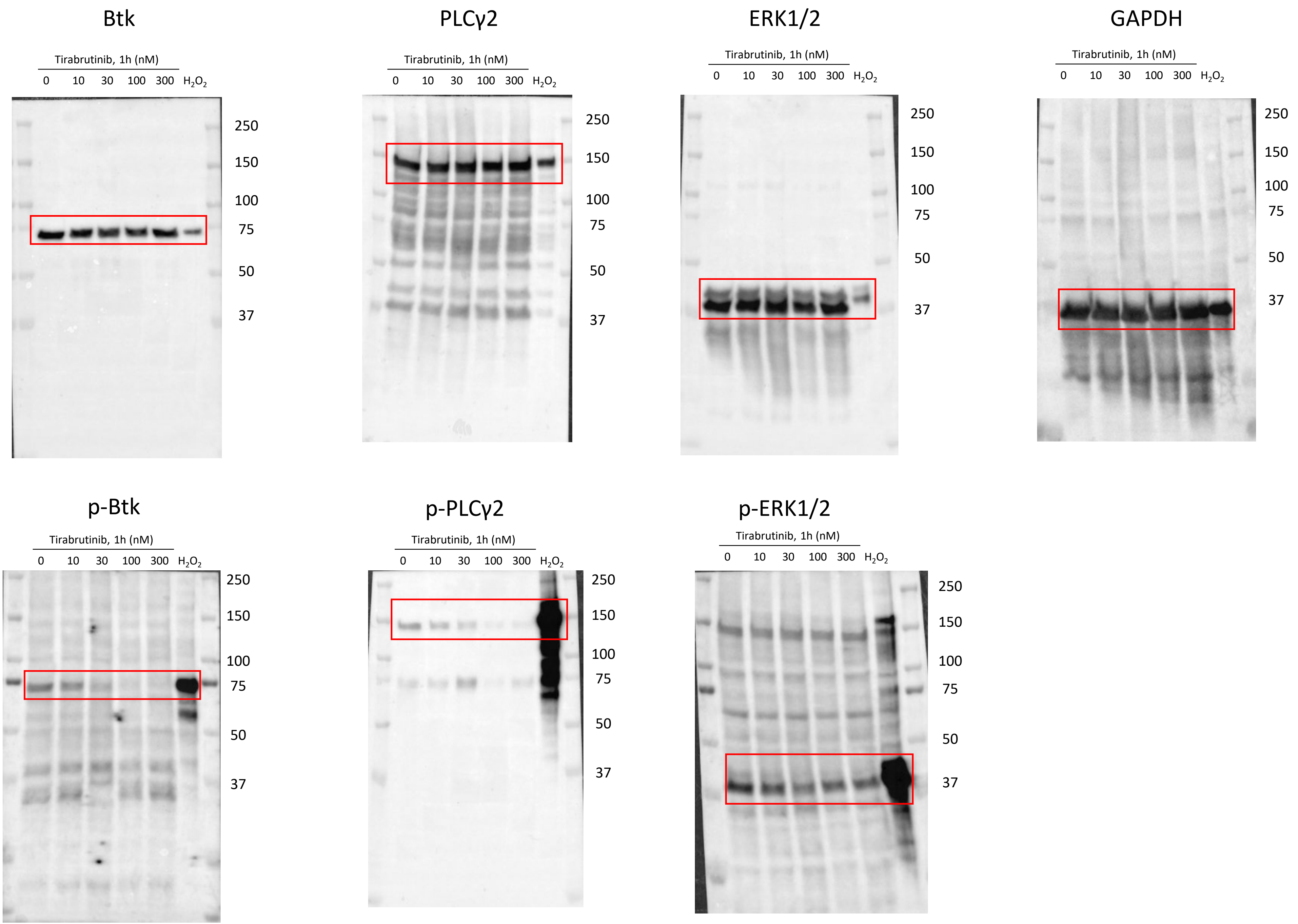

S2 Figure B_Original Image_Marker (U2932_Tirabrutinib_4 h)

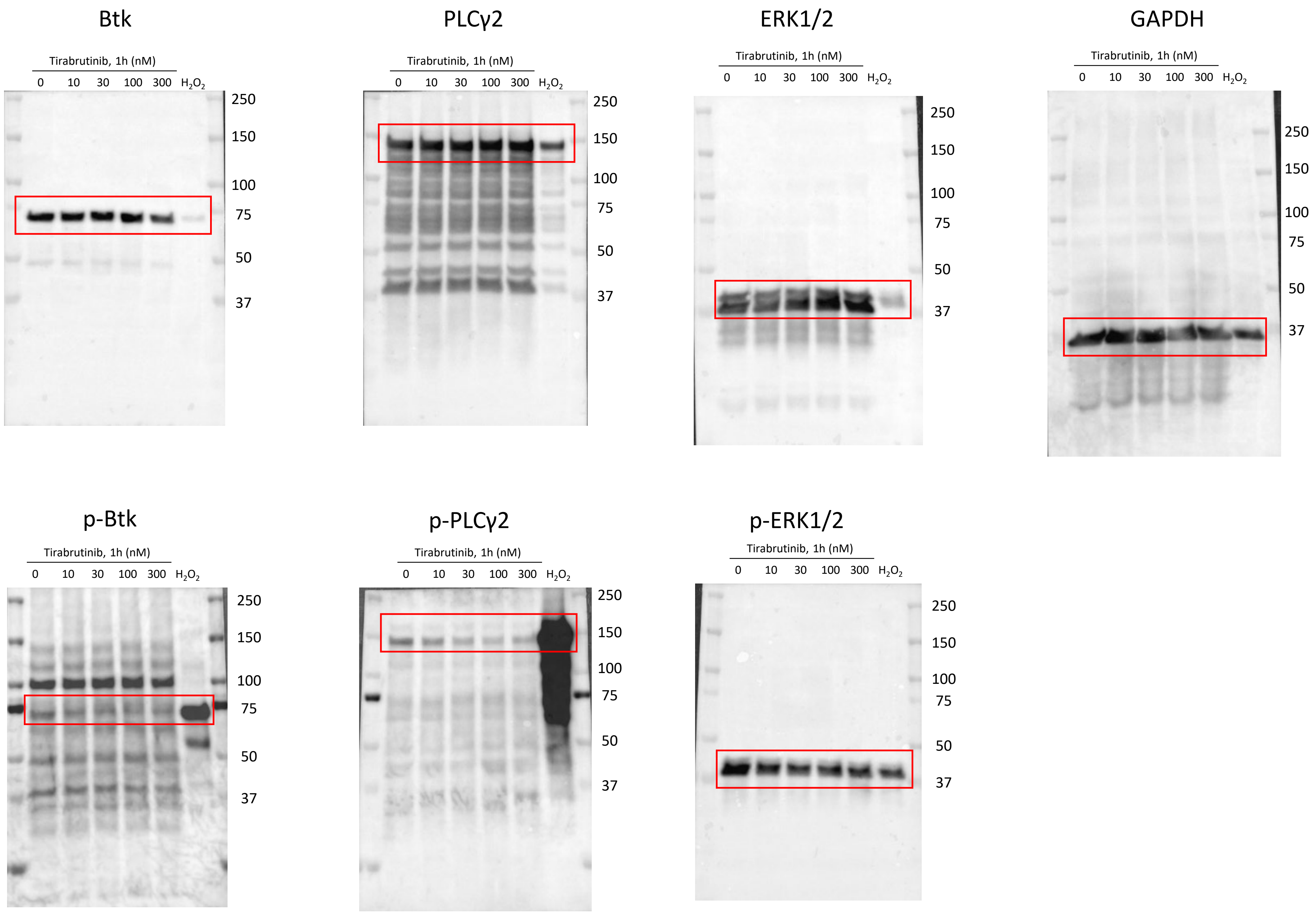

Supplement: S2 Raw images — (PDF) [file pone.0282166.s016.pdf]
