# Supplementary material for: Investigation of the anti-tumor mechanism of tirabrutinib, a highly selective Bruton’s tyrosine kinase inhibitor, by phosphoproteomics and transcriptomics
Source: PLoS One. 2023 Mar 10;18(3):e0282166. doi: 10.1371/journal.pone.0282166 (PMC10004634; doi:10.1371/journal.pone.0282166)
Supplement: Attachment — Submitted filename: Response to Reviewers.docx [file pone.0282166.s019.docx]

**Journal Requirements**

When submitting your revision, we need you to address these additional requirements.

1. Please ensure that your manuscript meets PLOS ONE's style requirements, including those for file naming. The PLOS ONE style templates can be found at

https://journals.plos.org/plosone/s/file?id=wjVg/PLOSOne_formatting_sample_main_body.pdf and

https://journals.plos.org/plosone/s/file?id=ba62/PLOSOne_formatting_sample_title_authors_affiliations.pdf

***Response****: We have ensured that our manuscript meets PLOS ONE's style requirements, including those for file naming.*

2. Thank you for stating the following in the Competing Interests section:

"I have read the journal's policy and the authors of this manuscript have the following competing interests: This study was funded by Ono Pharmaceutical Co., Ltd. (Osaka, Japan). All authors are employees of Ono Pharmaceutical Co., Ltd."

We note that you received funding from a commercial source: "Ono Pharmaceutical Co., Ltd."

Please provide an amended Competing Interests Statement that explicitly states this commercial funder, along with any other relevant declarations relating to employment, consultancy, patents, products in development, marketed products, etc.

Within this Competing Interests Statement, please confirm that this does not alter your adherence to all PLOS ONE policies on sharing data and materials by including the following statement: "This does not alter our adherence to PLOS ONE policies on sharing data and materials.” (as detailed online in our guide for authors http://journals.plos.org/plosone/s/competing-interests). If there are restrictions on sharing of data and/or materials, please state these. Please note that we cannot proceed with consideration of your article until this information has been declared.

Please include your amended Competing Interests Statement within your cover letter. We will change the online submission form on your behalf.

***Response****: We have provided an amended Competing Interests Statement and an amended Financial Disclosure in the cover letter.*

3. We note that you have indicated that data from this study are available upon request. PLOS only allows data to be available upon request if there are legal or ethical restrictions on sharing data publicly. For more information on unacceptable data access restrictions, please see http://journals.plos.org/plosone/s/data-availability#loc-unacceptable-data-access-restrictions.

In your revised cover letter, please address the following prompts:

a) If there are ethical or legal restrictions on sharing a de-identified data set, please explain them in detail (e.g., data contain potentially sensitive information, data are owned by a third-party organization, etc.) and who has imposed them (e.g., an ethics committee). Please also provide contact information for a data access committee, ethics committee, or other institutional body to which data requests may be sent.

b) If there are no restrictions, please upload the minimal anonymized data set necessary to replicate your study findings as either Supporting Information files or to a stable, public repository and provide us with the relevant URLs, DOIs, or accession numbers. For a list of acceptable repositories, please see http://journals.plos.org/plosone/s/data-availability#loc-recommended-repositories.

We will update your Data Availability statement on your behalf to reflect the information you provide.

***Response****: There are no ethical nor legal restrictions on sharing data. We have included the minimal anonymized dataset in Supporting Information as listed below:*

*S2 Table. Complete KINOMEscan dataset.*

*S3 Table. Processed version of the MaxQuant phosphosite table of the experiments with tirabrutinib-treated TMD8 cells.*

*S4 Table. Processed version of the MaxQuant phosphosite table of the experiments with tirabrutinib-treated U-2932 cells.*

*In addition, the microarray data are available from the Gene Expression Omnibus (GEO; GSE 210284). The other datasets used and/or analyzed during the current study are available from the corresponding author on reasonable request.*

4. PLOS ONE now requires that authors provide the original uncropped and unadjusted images underlying all blot or gel results reported in a submission’s figures or Supporting Information files. This policy and the journal’s other requirements for blot/gel reporting and figure preparation are described in detail at https://journals.plos.org/plosone/s/figures#loc-blot-and-gel-reporting-requirements and https://journals.plos.org/plosone/s/figures#loc-preparing-figures-from-image-files. When you submit your revised manuscript, please ensure that your figures adhere fully to these guidelines and provide the original underlying images for all blot or gel data reported in your submission. See the following link for instructions on providing the original image data: https://journals.plos.org/plosone/s/figures#loc-original-images-for-blots-and-gels.

In your cover letter, please note whether your blot/gel image data are in Supporting Information or posted at a public data repository, provide the repository URL if relevant, and provide specific details as to which raw blot/gel images, if any, are not available. Email us at plosone@plos.org if you have any questions.

***Response****: Original images of western blots have been provided as Supporting Information.*

**Responses to the comments of Reviewer #1**

In this work, Kozaki et al carefully characterized the selectivity of the BTK inhibitor, tirabrutinib, and its effects on phosphoproteomic in B-cell lymphoma cell lines. They also analyzed the transcriptomic changes induced by tirabrutinib in DLBCL xenograft tumors. Based on these results, the authors discussed the potential multifaceted anti-tumor mechanism of tirabrutinib. Overall, this manuscript provides interesting and important omics-data for the clinical BTK inhibitor, as well as potentially novel discoveries on the BTK signaling in DLBCL. However, I still feel that the current manuscript is not enough in-depth and need be further improved. Specifically, here are my major concerns:

1. The phosphoproteomic results should be further analyzed and sorted in terms of reported BTK substrates, reported or new direct/indirect downstream effectors, and potentially off-targets of tirabrutinib. For some important reported substrates or downstream effectors of BTK, the authors need to double-check with western blots if the proteomic studies failed to detect significant downregulation as expected, and then discuss it.

***Response****: We have sorted the phosphoproteomic analysis data regarding the reported BTK substrate PLCγ2 and reported or novel direct/indirect downstream effectors in* ***S5 Table****. However, we did not conduct additional analysis and sort any data for possible off-target candidates of tirabrutinib. The reason for this is as follows:*

*Based on the selectivity data using in vitro cell-free kinase assays, tirabrutinib at 1 μM inhibited kinase activity of TEC, BMX, HUNK, RIPK2, ERBB4, and CSK. Among these kinases, we have demonstrated that tirabrutinib did not bind to RIPK2 and CSK in a cell-based assay using PBMC (Kd; >3 μM), which was carried out using KinAffinity (Evotec AG, Munich, Germany). These results suggest that tirabrutinib would not inhibit RIPK2 and CSK in TMD8 and U-2932 cells. As for the other kinases (TEC, BMX, HUNK, and ERBB4), there are currently no publications that report their function or contribution to cellular signaling in DLBCL cells; thus, it is difficult to discuss the contribution of the potential inhibition of these kinases by tirabrutinib with the obtained phosphoproteomic data.*

*Next, we have investigated the effect of tirabrutinib on some important effectors, including the BTK substrate p-PLCγ2 and p-ERK, as well as p-BTK, by western blot analysis. The data were added as* ***S2 Fig****. In both TMD8 and U-2932 cells, tirabrutinib inhibited phosphorylation at Tyr-759, which is the site reported to be phosphorylated by BTK and correlates with the lipase activity of PLCγ2 [Mol Cell Biol. 2004;24:9986-99] at a similar concentration range to that required for the suppression of BTK autophosphorylation. This indicates that tirabrutinib inhibited the phosphorylation of PLCγ2 at Y759 through BTK inhibition, although significant suppression was not detected in the phosphosites of PLCγ2 in the phosphoproteomic analysis. Regarding p-ERK, we confirmed through western blot analysis that tirabrutinib inhibited p-ERK in TMD8 cells in a concentration-dependent manner after 1 h of treatment. In U-2932 cells, treatment with tirabrutinib for 4 h resulted in significant inhibition of p-ERK in a concentration-dependent manner, although the inhibitory activity of tirabrutinib on p-ERK was not clear during 1 h of treatment. Based on those results, we confirmed the inhibitory activity of tirabrutinib on p-ERK in both phosphoproteomic and western blot analyses.*

*As for another downstream signaling pathway, we also checked p-PKCβ and p-p65 in NF-κB signaling in both cells, and the data were added as* ***S3 Fig*** *in addition to the results for p-AKT, which were already submitted as supplemental data. Regarding PKCβ, a downstream effector of PLCγ2 and upstream effector of ERK and NF-κB signaling [Mol Cancer. 2018;17:57, Front Oncol. 2020;10:591577], in the phosphoproteomic analysis, tirabrutinib did not significantly inhibit the phosphorylation at Thr-641, which is the site that is reported to be autophosphorylated and that maintains catalytic competence of PKCβ. Therefore, we tried to detect p-PKCβ in the western blot analysis but failed in both cell lines because the tested antibody did not work well. Thus, it is difficult to conclude whether tirabrutinib has an effect on the phosphorylation of PKCβ. As for NF-κB signaling, several phosphorylated sites in the related effectors, such as IKKβ and p65, were detected in the phosphoproteomic analysis. However, it was difficult to determine whether tirabrutinib suppressed NF-κB signaling because the significance of those detected sites was unclear. In the western blot analysis, however, we successfully detected p-IκBα at S32/36, which is critical for the activation of NF-κB signaling [Biochem J. 2021;478:2619-2664.]. Because tirabrutinib inhibited p-IκBα in both cell lines, we concluded that tirabrutinib showed inhibitory activity on NF-κB signaling in both cell lines.*

2. For the downregulated transcriptions, the key factors also need be double-checked with qPCR and western blots. Besides, the authors should provide more proof that the downregulation of IRF4 and MYC w mainly induced by suppression of NF-κB in TMD8 cells. Moreover, it would be better if the authors could analyze the transcriptomic results together with the phosphoproteomic results, and check if there is consistence for factors such as ATF2 and ERF2.

***Response****: Because tirabrutinib downregulated IRF-4 and MYC pathway-related genes in the transcriptome analysis, we selected IRF4, BCL6, and MYC as key factors and evaluated the effect of tirabrutinib for expression levels of these genes and proteins after in vitro treatment with DMSO or tirabrutinib at 1 μM for 24 h in TMD8 cells by RT-PCR and western blot analysis. As a result, treatment with tirabrutinib resulted in downregulation of IRF4 and MYC genes, and upregulation of BCL6 genes, which was consistent with the microarray data. In the western blot analysis, we confirmed the downregulation of IRF4 and MYC protein expression by tirabrutinib, while a significant change in BCL6 protein was not induced by tirabrutinib. In conclusion, the results of IRF-4 and MYC pathway downregulation by tirabrutinib in the microarray experiment was confirmed with RT-PCR and western blot analysis. The obtained data and descriptions have been added to the Results section of the manuscript and* ***S6 Fig****.*

*Next, to clarify that the downregulation of IRF4 and MYC by tirabrutinib were mainly induced by suppression of NF-κB in TMD8 cells, we evaluated the effect of several drugs that selectively inhibit specific signaling pathways. As selective inhibitors for NF-κB, ERK, or AKT signaling, BMS-345541 (IKK inhibitor), refametinib (MEK inhibitor), and MK-2206 (AKT inhibitor) were used, respectively. As a result, treatment with BMS-345541 at 1 μM for 24 h resulted in significant downregulation in IRF4 and MYC genes. Treatment with MK-2206 at 1 μM did not downregulate these genes, and refametinib at 0.1 μM tended to downregulate only MYC genes. These results suggest that the effect of tirabrutinib on IRF4 would be mainly induced by suppression of NF-κB signaling, and the effect of tirabrutinib on MYC would be induced by suppression of not only NF-κB signaling but also ERK signaling. The obtained data and descriptions have been added to the Results section of the manuscript and* ***S7 Fig****.*

*Regarding your suggestion for analyses of the transcriptomic results together with the phosphoproteomic results, some descriptions were added to the Discussion section as follows:*

*“We also observed some correlations between the results of the phosphoproteomic and transcriptomic analyses of signaling pathways regulated by tirabrutinib in TMD8. For example, tirabrutinib significantly decreased the phosphorylation of ATF2 (Thr-71), which is phosphorylated by ERK, leading to transcriptional activation. ATF2 is reported to control ATF3 expression, which is an important element controlling the growth of ABC-DLBCL [66]. In the transcriptomic analysis, the expression of ATF3 was significantly suppressed by tirabrutinib (fold change, 0.36; P < 0.001). In addition, the observed downregulation at Thr-246 of PRAS40 by tirabrutinib in the phosphoproteomic analysis suggests the suppression of mTORC1 signaling [79], which is consistent with the downregulation of mTORC1 pathway-related genes by tirabrutinib in the transcriptomic analysis.”*

3. In Figure S1, the levels of native p-BTK were so weak that I doubt tirabrutinib inhibited p-BTK. The authors should check the phosphorylation of the direct substrate of BTK, PLCγ, and check if tirabrutinib could inhibit p-BTK after the H2O2 stimulation.

***Response****: As shown in* ***S2 Fig****, tirabrutinib inhibited p-BTK and p-PLC*γ*2 in a concentration-dependent manner in both TMD8 and U-2932 cells. The concentration at which tirabrutinib inhibits p-BTK is consistent with the data shown in* ***S1 Fig****. Additionally, tirabrutinib inhibited p-PLC*γ*2 (through Tyr-759, a phosphosite regulated by BTK) at a similar concentration range as for p-BTK, indicating that tirabrutinib inhibits the activation of BTK (p-BTK) in TMD8 and U-2932 cells. Furthermore, by flow cytometry, we confirmed that treatment with tirabrutinib for 6 h resulted in the inhibition of p-BTK in a concentration-dependent manner with an IC_50_ of 9.78 nM after H_2_O_2_ stimulation in TMD8 cells, as shown below (these data are not included in the manuscript).*

Figure: Effect of tirabrutinib on Btk inhibition by continuous exposure to TMD8 cells

*TMD8 cells were exposed to the vehicle or various concentrations of tirabrutinib for 6 h and then stimulated with H_2_O_2_. After stimulation, the cells were stained with BTK-pY223 antibodies, and the mean fluorescence intensity (MFI) of BTK-pY223 was measured by flow cytometry. From the MFI of each sample, the increase in MFI (ΔMFI) relative to that in the negative control sample was calculated, and the inhibition rates (%) in the tirabrutinib treatment groups relative to that in the vehicle group were displayed as the mean ± standard error for three samples per group.*

4. Tirabrutinib was much less potent to inhibit the growth of U-2932 cells, and downregulated much less phosphorylation of the proteome. The authors should further dig these findings, and check if U-2932 cells were really dependent on BTK as TMD8 cells, and look for the potentially difference of BTK signaling in both cells.

***Response****: As shown in* ***Fig 3A****, there is no significant difference in the IC_50_ of tirabrutinib for growth inhibition between TMD8 and U-2932, although the maximum growth inhibitions greatly differ in these cell lines. Tirabrutinib inhibited p-ERK at a similar concentration range to that of p-BTK inhibition (****S2 Fig****) and IC_50_ for growth inhibition (****Fig 3A, B****), which suggests that U-2932 cell growth is partially dependent on ERK signaling through p-BTK inhibition. Our phosphoproteomic and western blot analyses indicated that ERK signaling and NF-kB signaling are dependent on BTK in both TMD8 and U-2932 cell lines, whereas AKT signaling is not dependent on BTK in U-2932 cells, resulting in less downregulation of proteome phosphorylation and lower maximum growth inhibition in U-2932 compared with TMD8.*

And here are several minor concerns:

5. The abstract should be written more concisely, and the English in the main text should be further improved.

***Response****: The Abstract and main text have been revised by a native English speaker to address the Reviewer’s concerns.* *We have reduced the abstract word count to 208 words (note: the PLOS ONE Abstract limit is 300 words).*

6. The culturing density and the type of well plates should be added into the method for the cell viability assays.

***Response****: We have added information about the culturing density and well plates used to the Methods section.*

7. Despite that tirabrutinib showed overall consistent IC50s regarding inhibition of cell growth and phospho-BTK, more evidence is needed to support the conclusion that “proliferation appears to be inhibited by tirabrutinib via inhibition of BTK phosphorylation” in line 393. Besides, tirabrutinib could not suppressed the growth of U-2932 cells even at high concentrations.

***Response****: Please see our response to comment #4. Tirabrutinib inhibited p-ERK at a similar concentration range to that of p-BTK inhibition (****S2 Fig****) and IC_50_ for growth inhibition (****Fig 3A, B****) in TMD8 and U-2932 cells. These data suggest that tirabrutinib exerts an anti-proliferative effect via p-ERK inhibition by p-BTK inhibition in both cell lines. Regarding the reason for the difference between TMD8 and U-2932 in the maximum inhibition for cell growth, we speculate that AKT signaling is not dependent on BTK in U-2932.*

8. In line 478, the description “although the significances of all the regulated sites by tirabrutinib are unknown” is a bit confusing. Did the authors mean the functions of these phosphorylations?

***Response****: Yes, we were referring to the functions of all the regulated phosphosites. We have revised the description in the Results section to avoid confusion.*

**Responses to the comments of Reviewer #2**

Reviewer #2: In the manuscript of "nvestigation of the anti-tumor mechanism of tirabrutinib, a highly selective Bruton’s tyrosine kinase inhibitor, by phosphoproteomics and transcriptomics" , the study is well defined. The flow of work is good starting from showing the selective of this drug on BTK among other kinases to in vivo test the efficacy of this drug.

1. It would be good if the author can show the structure of the complex between three generation of drugs with BTK and explain the reason why it selective against BTK than other kinases.

***Response****: Based on the Reviewer’s suggestion, we have added the following description in the Discussion section:*

*“The binding of zanubrutinib to BTK is different from that of ibrutinib to BTK, which contributes to its superior selectivity [14]. The binding mode of tirabrutinib and acalabrutinib with BTK is similar to that of ibrutinib, but the structures of the inhibitory component are different. Butylamide electrophiles are less reactive than acrylamide, and the higher nucleophilicity of Cys-481 in BTK may explain the superior selectivity over ibrutinib [13].”*

**Additional revisions**

*In addition to the revisions outlined above, we have also made some minor amends of the text to correct some inconsistences in terminology and typographical marks. These changes are indicated using Microsoft Word’s Track Changes.*
